# Attending lectures in person, hybrid or online—how do students choose, and what about the outcome?

**DOI:** 10.1186/s41239-023-00387-5

**Published:** 2023-03-28

**Authors:** Gerd Kortemeyer, Nora Dittmann-Domenichini, Claudia Schlienger, Ekkehard Spilling, Alina Yaroshchuk, Günther Dissertori

**Affiliations:** 1 Educational Development and Technology, ETH Zurich, Haldenbachstrasse 44, 8092 Zurich, Switzerland; 2 Department of Physics and Astronomy, Lyman Briggs College, Michigan State University, 426 Auditorium Road, East Lansing, MI 48824 USA; 3 Rectorate, ETH Zurich, Rämistrasse 101, 8092 Zurich, Switzerland

**Keywords:** Attendance modes, Hybrid teaching, Online teaching, On-site teaching, Recordings, Self-determination

## Abstract

As a consequence of the COVID-19 pandemic, most courses at a large technical university were adapted so that students had a free choice of whether to attend lectures on-site or online; in addition, in many courses, lecture recordings were available. At the subsequent exam session, over 17,000 student-survey responses were collected regarding attendance choices, learning behavior, interest in the course, perception of the exam, and recommendations to future students. A total of 27 learner attributes and their relationships were investigated. In addition, conditional attributes and free-response statements were analyzed, and the students’ exam grades were retrieved to gauge their performance. We found only minute differences with respect to exam performance, but the analysis indicates distinctly different preferences and constraints in taking advantage of learning opportunities. We also found some indications that performance differences might be larger for interactive-engagement courses. The results of the analysis may be key to answering why at many universities, faculty report that live-lecture attendance has decreased more strongly than expected with the availability of new, virtual attendance modes.

## Introduction

The COVID-19 pandemic has occasionally been viewed as one of the biggest experiments in education (Thomas & Rogers, 2020; Dunrong & Jin, 2020). This might be a misnomer, since “experiment” implies some sort of controlled conditions, while arguably, educational settings were largely controlled by fluctuating, external factors. “Disruption” might be a more fitting characterization of what was essentially an emergency response, and in the aftermath of this disruption, increased flexibility in attendance and delivery modes of education will become the “new normal” (Kortemeyer, 2020; Schapiro, 2021; Hofer et al., 2021). The educational experiment starts now, as the impact of this flexibility can be investigated in more controlled settings. A preliminary “finding” of this experiment is that many faculty members report that live-lecture attendance has decreased—some faculty members even go so far as to demand that streaming, video conferencing, and recording should be discontinued, “now that the pandemic is over,” to force students to return to campus. There might be some justification for that: both students and faculty who knew the university before COVID-19 bemoan the loss of campus culture, and there are certainly cross-disciplinary and social competencies that were implicit in higher education, such as scientific discourse, self-presentation, teamwork, conflict resolution, etc., which may not be fostered anymore when purely focussing on the explicit curriculum of teaching and transmitting facts, methods, and concepts. There are also serious concerns about loneliness, depression, anxiety, and procrastination that need to be addressed (Wang et al., 2020; Pelikan et al., 2021; Copeland et al., 2021; Tasso et al., 2021; Amendola et al., 2021; Buizza et al., 2022), which are consistent with a survey on student well-being conducted at ETH Zurich at the height of the pandemic. The problems and their solutions are likely more complex and reaching deeper—the pandemic may have simply brought some existing inconsistencies in the 21st-century higher-education system to the surface, particularly when it comes to lecturing (Vlachopoulos & Jan, 2020).

An immediate question is how student choices regarding attendance may have influenced performance in the subsequent exam session. Finally, throughout the whole pandemic, high-stake exams were conducted in-person on-site at ETH Zurich, and another question is how the students’ perception of these physical exam settings may be connected to their potentially completely virtual attendance during the learning phase.

## Setting

ETH Zurich is a large, technical university, which generally appears prominently in international rankings. It places heavy emphasis on research across a wide spectrum of STEM disciplines, but also offers architecture and humanities. The university has around 25,000 students from 120 countries, about a third of which are female (ETH, 2023).

The study presented here took place during the exam period of Spring semester 2022, at what is assumed to be the tail end of the pandemic. In most courses at ETH Zurich, due to the pandemic, students had a choice of how to attend the Fall 2021 and Spring 2022 courses leading up to these exams: on-site, live online, or simply by watching recordings. Assuming that a survey of 639 courses conducted in a later semester at ETH Zurich is representative, in spite of technology-mediated attendance options, the majority of the courses in this study would likely have been delivered predominantly in traditional frontal-lecture style—only a small percentage of faculty self-reported frequently employing interactive-engagement strategies such as the use of audience response devices (“clickers”) (MacGeorge et al., 2008; Hunsu et al., 2016). At the same time, the university prides itself in providing meaningful project work, and it is actively fostering the establishment of more opportunities and infrastructure for curricular and extracurricular projects.

ETH Zurich has some peculiarities, shared with some other European universities, that may influence responses. While basically all of the exams cover particular courses, technically they are separate from these courses: a student can be enrolled in a course and not take the exam (or take it after a later semester). During the running semester, no high-stake assessments, i.e., assessments that have influence on the ability of continuing a study program, are allowed; any assessment can at most count as a limited number of bonus points toward the subsequent high-stake exam. There are both end-of-semester and so-called session exams, the latter of which take place each semester after the summer or winter holidays, respectively. If students do not pass an exam, they can repeat it once; depending on the course, this can happen with or without having to enroll again in the course. Finally, there are year-long courses which are covered with one large, high-stake exam at the end—especially, the first year examinations of most bachelor-degree programs are held after two semesters of basic studies. Thus, it can happen that students are tested on the concepts and contents of a particular course more than one semester later, i.e., after an intermittent semester and the summer holidays. Students also frequently work on projects (design projects, thesis projects, and course assignments) in laboratories or student-led construction spaces [“maker spaces” (Barrett et al., 2015)], as well as in fieldwork.

The enrollment system allows students to enroll in courses that happen at the same time in different places. Enrolling in a large number of courses (the limit is 15) does not come with a financial penalty, as credits are free-of-charge.

Located in an urban setting, ETH Zurich also has two main campuses and some courses taking place at satellite locations, as well as rooms on the campus of another university, where courses take place. Zurich consistently ranks among the top-10 cities worldwide with the highest cost of living; there is an insufficient number of student dormitories, which are privately owned. Still, in an internal survey conducted by ETH Zurich’s department of Student Services in 2021, 86% of the students stated that their financial situation allowed them to allocate sufficient time for their studies; of the remaining 14%, 80% reported that this is due to having to earn money working—so, by implication, 11% of the students could not allocate enough time for their studies due to the need for working in a job.

Like essentially all other universities, in Spring 2020, ETH Zurich had to switch to emergency teaching from one week to the next. Overall, as far as carrying on the teaching mission of the university is concerned, this worked surprisingly well. In two separate ETH-internal surveys commissioned by the rectorate in Spring 2020 and Fall 2020, only about 10% of the students stated that remote teaching did not or not really work for them; unfortunately, about the same percentage stated that thus they were not or not really able to keep up with the materials. By the reverse token, in Spring over 40% of the students stated that remote teaching worked really well for them, and that they were able to really well keep up with the materials—those numbers, however, unfortunately deteriorated in the Fall to about 30% and 20%, respectively, where students mostly downgraded their assessment of remote teaching from working really well to working well. Comparing student cohorts starting as early as Fall 2017 to later cohorts starting at the onset, during, and at the tail end of COVID-19 showed that year-to-year attrition and success rates were not affected by remote teaching.

All high-stake exams, including the ones in this study, were carried out on-site under supervision, even at the height of the pandemic. Hygiene protocols, crowd-control, and distancing measures were put in place to ensure both the safety of students, faculty, and staff, and the integrity of the exams [one of the largest challenges during and in the aftermath of COVID-19 (Hwee et al., 2022)].

## Methods

### Ethics statement

Human subject research approval was obtained as protocol ETH Zurich EK 2022-N-137. Informed consent was obtained from all participants in this study.

### Data collection

The exam evaluations (Dittmann-Domenichini et al., 2015) during the exam session following the spring semester 2022 were expanded by questions on preparatory learning behavior. Answering these additional questions was voluntary, as explained in the introductory section of the survey. Filling out those questions indicated consent for participation in this study, as per human-subject research protocol explained in the same introduction. For participating students, data from the central administrative system was linked.

Students received one survey per exam, so, by extension, each survey represents one student-course enrollment; there were a total of 43,979 such surveys given out. The same student may have filled out more than one survey and may have done so differently for different courses that they might have attended and studied for in different ways. A total of 17,641 surveys covering 381 courses indicated agreement for participation in the study. Of those, 13,585 filled out the survey completely with responses to all items. Not all courses included recordings, but 16,476 surveys also included data on usage of recordings—of those, 12,939 surveys were filled out completely.

A concern was that the survey might only reach students for whom online forms of teaching during the pandemic worked, while students for whom online did not work well would have dropped out or not registered for the exams. In essence, due to the preceding pandemic, there would have been a selection bias towards students who prefer online over face-to-face teaching. However, we believe this not to be the case, based on the Spring 2020 and Fall 2020 surveys, as well as the data on student attrition and success rates, which have been consistent across cohorts well before, during, and after the pandemic.

### Considered student attributes

A total of 27 attributes were considered for the analysis, shown in Table 1. These can be roughly divided into perceptions of the on-site exam conditions, the preparation that the course and its materials provided for the exam, the way in which the learners studied for the exam (including the two negatively formulated statements *LearnMinEffort* and *LearnMorePos*), ways of lecture attendance, and data gathered from the administrative systems. In addition to the Likert scales, many questions included a “non-applicable”-type choice, e.g. “no recordings”.

**Table 1:** The labels, descriptions, and ranges of the attributes considered in this study

Label	Description	Range
*CanExplain*	“I could explain the concepts to fellow students”	1 (do not agree)–5 (completely agree)
*CoursePrepared*	“The course prepared me for the exam”	1 (do not agree)–5 (completely agree)
*CourseSatisfaction*	“I was satisfied with the course”	1 (do not agree)–5 (completely agree)
*EstimatedGrade*	Student-estimated grade on the exam	1.0 (worst)–6.0 (best)
*ExamAlignment*	“Knowledge tested and level required were aligned with teaching”	1 (do not agree)–5 (completely agree)
*ExamCorresponds*	“How much did the exam correspond to your expectations?”	1 (not at all)–5 (completely)
*ExamEmphasis*	“The exam emphasized ...”	1 = facts; 2 = connections; 3 = applications
*ExamEnviron*	“The exam environment was appropriate”	1 (do not agree)–5 (completely agree)
*ExamFairness*	“The exam was fair”	1 (do not agree)–5 (completely agree)
*ExamFormulation*s	“Formulations on the exam were clear”	1 (do not agree)–5 (completely agree)
*ExamGrade*	Actual grade on the exam (from administrative system)	1.0 (worst)–6.0 (best)
*ExamSameCond*	“During the exam, everyone had the same conditions”	1 (do not agree)–5 (completely agree)
*ExamTime*	“The time for the exam was ...”	1 (way to short)–3 (reasonable)
*FacComExpect*	“Faculty clearly communicated expectations”	1 (do not agree)–5 (completely agree)
*Gender*	Gender (from administrative system)	1 = male; 2 = female
*LearnConnect*	“I tried to connect the topics and my previous knowledge”	1 (do not agree)–5 (completely agree)
*LearnEnjoy*	“I enjoyed learning the topics”	1 (do not agree)–5 (completely agree)
*LearnGroup*	“I learned in groups”	1 (never)–5 (frequently)
*LearnMinEffort*	“I only invested minimal effort”	1 (never)–5 (frequently)
*LearnMorePos*	“I could have studied more intensively”	1 (never)–5 (frequently)
*LearnPersist*	“If I encountered difficulties with a topic, I did not give up”	1 (do not agree)–5 (completely agree)
*LiveCampus*	“I attended lectures live on campus”	1 (almost never)–5 (always)
*LiveOnline*	“I attended lectures live online”	1 (almost never)–5 (always)
*MatPrepared*	“The course materials prepared me for the exam”	1 (do not agree)–5 (completely agree)
*PriorInterest*	“I was interested in the topics before the course”	1 (do not agree)–5 (completely agree)
*RecOnly*	“I only watched the recording instead of attending lecture”	1 (almost never)–5 (always)
*RecPrepared*	“The recordings prepared me for the exam”	1 (do not agree)–5 (completely agree)

The descriptions are paraphrased here in the interest of conciseness

The survey also included conditional questions, such as “Why did you prefer watching only the recordings?” if students indicated they at least frequently did so. For example, the conditional question on recordings also provided some choices for anticipated reasons based on feedback from student representatives, a free-response field for other reasons, and a follow-up question if the respondent would recommend for other students to do the same. These conditional attributes are listed in Table 2. For free-response questions, in a later step, themes were found and the answers categorized to enable quantitative statements.

**Table 2:** The labels, conditions, descriptions, and ranges of attributes considered in this study that were only surveyed depending on other attributes

Label	Condition	Description	Range
*NotLiveCampReason*	*LiveCampus* < 50%	“Why did you rarely or almost never attend lecture live?”	Free-response
*RecOnlyWhy*	*RecOnly* ≥ 50%	“Why did you prefer watching only the recordings?”	Fit better timewise; could learn better that way; had another lecture at the same timeslot; no commuting; other reasons
*RecOnlyReason*	*RecOnlyWhy* = other reasons	“What other reasons?”	Free-response
*RecRecom*	*RecOnly* ≥ 50%	“Would recommend only watching the recordings?”	Yes/no

The descriptions are paraphrased here in the interest of conciseness

### Considered course attributes

Unfortunately, little data was available concerning how the courses were conducted. Based on the independently conducted faculty-survey regarding instructional activities, within our data, 13 courses with a total of 446 survey responses could be identified where instructors self-reported frequent use of clickers. Clickers here are used as a proxy for interactive-engagement, as they are frequently also associated with peer instruction (Crouch & Mazur, 2001; Vickrey et al., 2015; Riegler, 2019). The clicker app used at ETH Zurich, EduApp (Gunnar, 2018; Aka et al., 2020), allows for participation in-class and online.

### Analysis

Much of the remaining analysis was carried out similarly to a related study at another university (Kortemeyer et al., 2022). In particular, exploratory cluster analysis (R Core Team, 2018; Golino, 2022) and Fruchterman-Reingold (Fruchterman & Reingold, 1991; Bastian et al., 2009) representations based on cosine similarities (Watanabe & Cannoodt, 2022) between student-attribute vectors were used to identify the relationships between attributes.

## Results

### Attendance modes

**Fig. 1:**
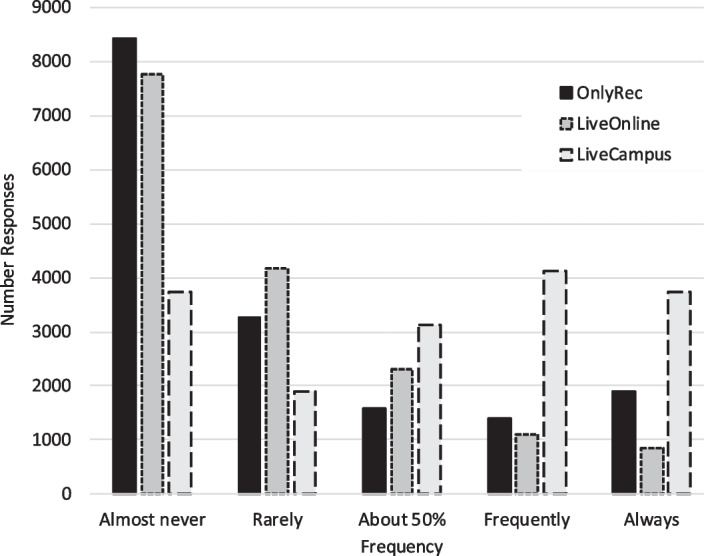
Number of students versus frequency of attendance on campus (*LiveCampus*), attendance online (*LiveOnline*), and only watching the recordings (*RecOnly*)

Figure 1 shows how students took advantage of the flexibility in attendance modes.

There is a large number of responses indicating that electronic options of following the course were never used; these roughly correspond to the sum of the responses that indicate attending lectures on campus frequently or always. The number of responses indicating extensive high reliance only on recordings is comparatively low, and so is the number of responses indicating more than 50% usage of live online transmissions.

To get a rough estimate of overall usage, one could arbitrarily set “rarely” to $\frac{1}{4}$th and “frequently” to $\frac{3}{4}$th of the time. This would lead to a *RecOnly*-usage of 26%, a *LiveOnline*-usage of 22%, and a *LiveCampus*-usage of 50%. In this estimate, the remaining 2% would correspond to skipping lectures altogether. This corresponds to the observation by some instructors that lecture halls were about half as full as expected.

The number of responses indicating mixed behavior (“about 50%”) is small but still considerable. The data, unfortunately, does not indicate if these students mixed attendance modes throughout the semester (for example, between different days of the week), or if they at some point during the semester decided to switch from one mode to the other.

### Attendance versus exam grades

**Fig. 2:**
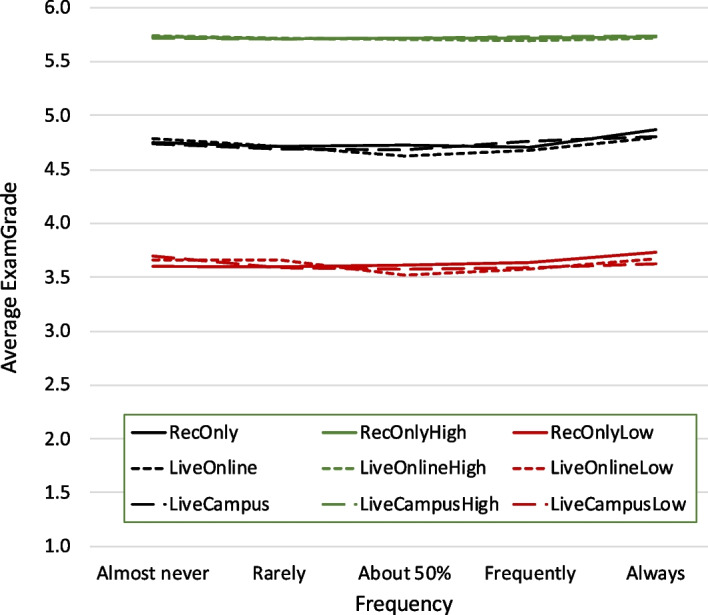
Average grade on exams (*ExamGrade*) versus frequency of attendance on campus (*LiveCampus*), attendance online (*LiveOnline*), and only watching the recordings (*RecOnly*). Shown in black are the average exam grades for all students, shown in green and red are the performances of the upper and lower quartile, respectively

The average *ExamGrade* of all survey responses is $4.7\pm 0.8$ (compared to $4.6\pm 0.9$ for all exams). An immediate question is whether or not the attendance choices have any influence on the exam grades; as Fig. 2 shows, this is essentially not the case—there are only minimal differences in average exam performance between the frequencies of attendance in different delivery modes. Shown in addition to the overall performance (black lines) are the averages of the upper (green) and lower (red) quartile of the exam grades—while it is common lore that high-performing students will likely succeed independent of educational scenario, at least when it comes to attendance modes, this independence is also evident for the lower-performing students.

There is definitely no straightforward dependency along the lines of “the more students go to live lectures, the better their grade”—if anything, the averages are worse for students who switch between attendance modes. For example, students who attend live lectures 50% of the time on the average have exam grades that are 0.12 lower than students who always and 0.06 lower than students who never go to live lectures on campus; intriguingly, these minimal differences are statistically significant according to Welch’s ANOVA at $p<0.001$. In other words, students who have not clearly settled into one attendance mode do significantly worse, but this significant difference is around 0.1 out of 5.0.

**Fig. 3:**
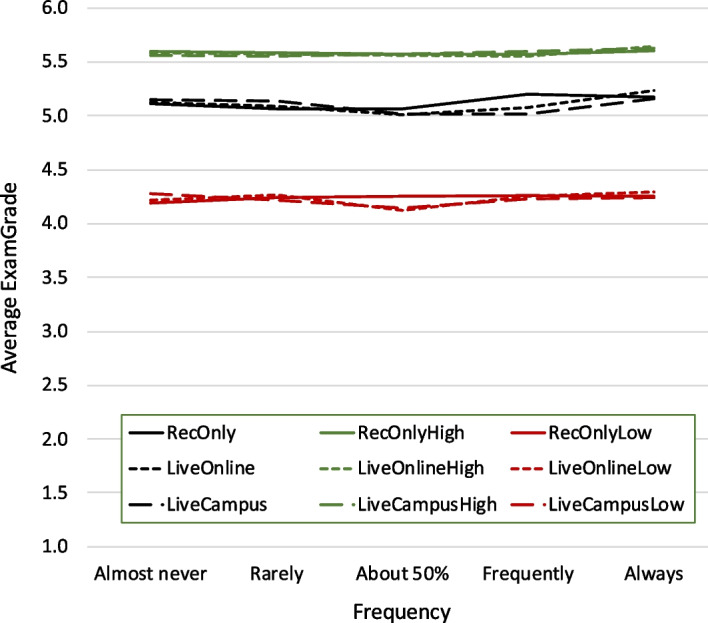
Same as Fig. 2, but only for courses with an average *CoursePrepared* and an average *CourseSatisfaction* above “agree”

Another assumption could be that there is a stronger attendance-mode effect for courses that were rated particularly high by students, that is, for courses where attendance might have a bigger impact. Figure 3 shows the same as Fig. 2, but limited to courses with an average *CoursePrepared* and an average *CourseSatisfaction* above “agree” (1632 responses covering 42 courses). While exam grades are generally higher in these highly rated courses, also here there is no straightforward dependency on attendance modes.

**Fig. 4:**
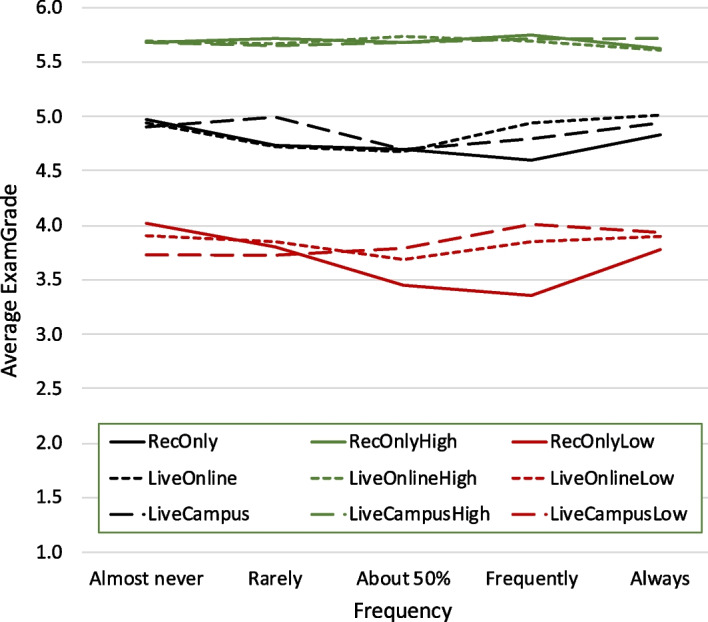
Same as Fig. 2, but only for courses whose instructors indicated frequent use of clickers on a separate faculty survey. The data originates from 13 courses and 446 survey responses. The observed fluctuations, however, are statistically non-significant ($p>0.05$)

We also considered interactive-engagement courses, where usage of clickers was used as a proxy. Figure 4 shows the same as Fig. 2, however, only for these interactive-engagement courses. While the general trends indicate that grade differences are larger for these courses (over 0.5 points), particularly for the lower-achieving students, the fluctuations are statistically non-significant ($p>0.05$).

**Fig. 5:**
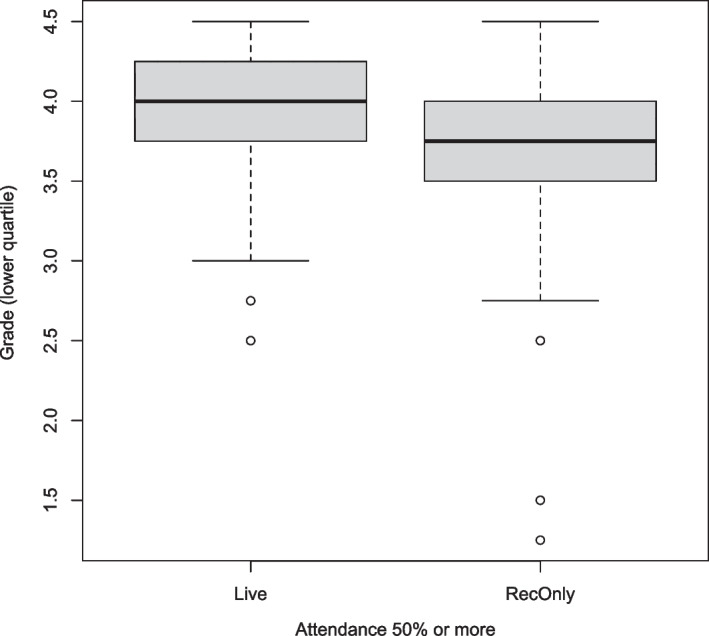
Boxplot illustrating grade differences of students in the lower performance quartile who followed interactive-engagement lectures live 50% or more of the time versus students in the lower performance quartile who only watched the recordings 50% or more of the time

Comparing students in the lower performance quartile who 50% or more attended lectures live (combining *LiveCampus* and *LiveOnline*) to students from the same quartile who 50% or more only watched the recordings does show a moderately significant difference (Welch’s ANOVA yielding $p<0.05$); this is also illustrated in Fig. 5. These significant differences suggest that live attendance of interactive-engagement lectures can make a significant difference.

Finally, there were 738 survey responses which stated that lectures were neither attended nor watched, i.e., *LiveCampus* = *LiveOnline* = *RecOnly* = “almost never”. The average *ExamGrade* for these respondents who skipped lectures altogether was $4.6\pm 0.8$, which is essentially indistinguishable from the overall average.

### Attribute-cluster analysis

**Fig. 6:**
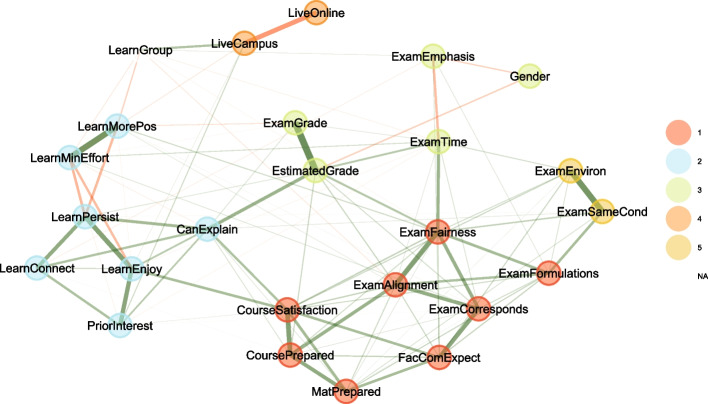
Exploratory cluster analysis between the student attributes in Table 1 for all courses, regardless of the availability of recordings (13,585 completely filled out surveys; attributes related to recordings are not part of this analysis, since not all students could have provided them). The edges indicate the correlations between the attributes, where negative correlations are indicated in red, while positive correlations are green; the thickness of the edges indicates the absolute values of the correlations. The colors indicate clusters found in an exploratory analysis

**Fig. 7:**
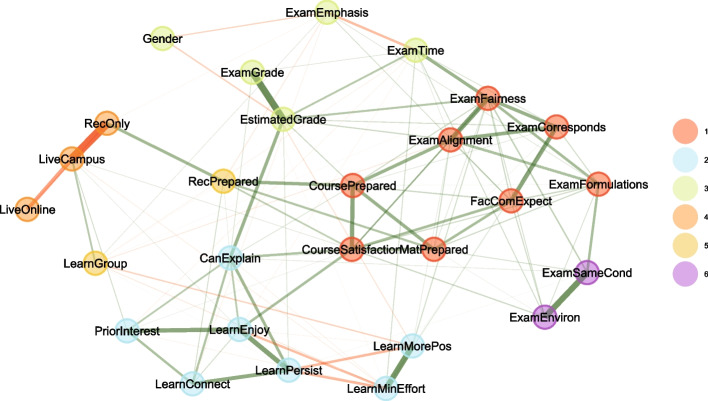
Same as Fig. 6, but only for courses that included recordings (12,939 completely filled out surveys), and including the corresponding attributes

Carrying out an exploratory cluster analysis shows how the attributes are correlated to and connected with each other. Figure 6 shows a graphical representation of the correlations and detected clusters based on all courses, and Fig. 7 shows the same only for courses that offered recordings (note that the rotation and handedness of the graphs are random).

Immediately noticeable is that the attributes related to attendance modes form a cluster that is separate from all other attributes (Cluster 4 in Figs. 6 and 7). This cluster is weakly positively correlated via *LiveCampus* to *LearnGroup*—students who come to campus are more likely to study in groups—and also weakly positively correlated to *PriorInterest*—students who have a prior interest in the topic are more likely to come to campus. For courses that offered recordings, the cluster is moderately positively correlated via *RecOnly* to *RecPrepared*—students who mostly only view recordings are more likely to state that they prepared well for the exam. As *LiveCampus* and *RecOnly* strongly negatively correlate, this also explains why *LearnGroup* and *RecPrepare* form a cluster (Cluster 5 in Fig. 7; there is no counterpart in Fig. 6, where *LearnGroup* is unassociated): one could surmise that students who come to campus might feel that they would best prepare for exams in groups, while students who only watch the recordings feel that this prepares them individually.

Some other clusters, not related to attendance modes, are of possible general interest:

- Cluster 1 joins exam expectations and alignment with course features; in essence, this cluster consists of attributes how well the exam and the course matched up. Interestingly, this cluster is not strongly linked to exam performance.
- Cluster 2 describes learning behaviors (except *LearnGroup*). A link to other clusters is provided by *CanExplain*: it is linked to both the estimated performance on the exam (*EstimatedGrade* from Cluster 3) and the satisfaction with the course (*CourseSatisfaction*) and thus probably one of the best indicators of successful and enjoyable education: the ability to explain the material to others (“peer-teaching”) plays a central role.
- Cluster 3 contains the estimated and actual grade on the exam. These two are strongly positively correlated, meaning, students can estimate their own performance fairly well. Somewhat surprisingly, *Gender* is part of the same cluster, but mostly due to the weakly negative correlation to *EstimatedGrade*, while there is only a minimal correlation to the actual *ExamGrade* (women on the average achieve 0.1 exam points less than men (Welch’s ANOVA estimates $p<0.001$)). Women apparently tend to underestimate their performance, while men tend to overestimate.
- Cluster 5 in Fig. 6, which is Cluster 6 in Fig. 7, describes external conditions of the exam. Naturally, it is linked to *ExamFairness* and *ExamFormulations*, but not to the grades.

### Response-cluster analysis

Based on earlier studies, it was suspected that *LiveCampus* would be a dominating attribute when it comes to similarities between survey responses within courses that had recordings (Fig. 7). Their attribute-values (Table 1) were interpreted as a feature vector, normalized, and the cosine similarities between them were interpreted as edges in a graph. A Fruchterman-Reingold representation of this graph confirmed that survey responses align along the *LiveCampus*-attribute.

Investigating the influence of this particular attribute on other attributes, data on attributes were converted to a binary format based on being above or below average, and Fisher’s Exact Test was carried out on the contingency tables to determine the likelihoods of other attributes being above average for students who visited live lectures more than the average student. Table 3 shows the statistically significant results.

**Table 3:** Statistically significant results of Fisher’s Exact Test on attributes being above average for students who went to live lectures more frequently than the average student

Label	Odds ratio	*p*-value
*CanExplain*	1.1	$p<0.001$
*CoursePrepared*	1.1	$p<0.001$
*CourseSatisfaction*	1.3	$p<0.0001$
*ExamCorresponds*	0.9	$p<0.01$
*ExamEmphasis*	1.1	$p<0.05$
*ExamEnviron*	0.9	$p<0.05$
*ExamGrade*	1.1	$p<0.01$
*ExamTime*	0.9	$p<0.05$
*LearnEnjoy*	1.4	$p<0.0001$
*LearnConnect*	1.2	$p<0.001$
*LearnGroup*	1.8	$p<0.0001$
*LearnMinEffort*	0.7	$p<0.0001$
*LearnMorePos*	0.7	$p<0.0001$
*LearnPersist*	1.3	$p<0.0001$
*LiveOnline*	0.4	$p<0.0001$
*PriorInterest*	1.5	$p<0.0001$
*RecOnly*	0.04	$p<0.0001$
*RecPrepared*	0.7	$p<0.0001$

The Odds Ratio describes how much more likely the attribute is above average when the *LiveCampus* one is, it does not imply causation. An Odds Ratio of 1.0 means no difference. Students who visited live lectures more frequently have above-average confidence in their exam preparation, but also seem to expect a little more from the exam and its environment: they are $1/0.7\approx 1.4$-times less likely to make an above-average statement that they only put in minimal effort, but are also $1/0.9\approx 1.1$-time less likely to state that they had above-average enough time or an above-average exam environment. They are more likely convinced of their choice not to have relied on the videos. In the end, though, going to live lecture more frequently than average only makes it 1.1-times more likely to get an above-average grade.

### Retrospective assessment of attendance choices

It turned out that students did not distinguish between the responses to *NotLiveCampReason*, *RecOnlyWhy*, and *RecOnlyReason* (see Table 2); instead, several respondents referred from one answer to the other, e.g., they wrote, “please see answer above,” and they generally commented on why they would not attend live lectures on campus. It was thus decided to combine those answers.

**Fig. 8:**
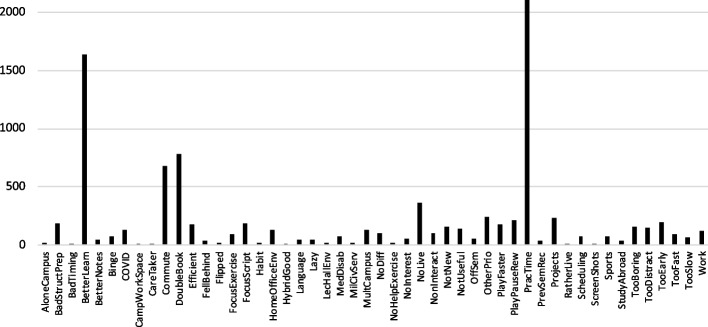
Frequencies of the categories in Table 4

A total of 6412 surveys had entries for one or more of these attributes, which were ad-hoc classified. Table 4 shows the established categories, and Fig. 8 shows their frequency. The most frequently named reason for not attending live lecture is that it was more practical time-wise, and the second-most frequent reason is that one could learn better that way. Somewhat surprisingly, having more than one lecture at the same time (*DoubleBook*) was mentioned more frequently than the commute to campus (*Commute*). Some students voluntarily double-booked and overloaded their schedule, while others expressed that they had no choice due to the layout of their curriculum. As one student explains:

“I had 10 or 11 classes scheduled this semester per the study plan, which meant I could not attend all subjects 100% of the time.”

“I attended 3 lectures at the same time. But was no problem and always watched the lecture afterwards.”

—in any case, allowing such double (and apparently also triple) bookings within the enrollment system might have prevented curricular conflicts from surfacing.

**Table 4:** Categories of responses to items *NotLiveCampReason*, *RecOnlyWhy*, and *RecOnlyReason*

Label	Description
*AloneCampus*	Did not know fellow students, would have felt alone on campus
*BadStructPrep*	Lecture was badly structured or ill-prepared
*BadTiming*	The timing of the materials over the course of the semester was not optimal
*BetterLearn*	Could learn better outside the lecture hall
*BetterNotes*	Could better take note outside the lecture hall
*Binge*	Would watch all lecture recordings back-to-back
*COVID*	Ongoing COVID-19 situation
*CampWorkSpace*	Watched on-campus, but outside the lecture hall
*CareTaker*	Had to take care of relatives or pets
*Commute*	Had to commute long-distance to campus
*DoubleBook*	Had another lecture at the same time
*Efficient*	Learning was more efficient outside the lecture hall
*FellBehind*	Fell behind in the course and could not follow live lectures anymore
*Flipped*	Course was taught in a flipped-classroom mode
*FocusExercise*	Focussed on the exercises
*FocusScript*	Focussed on the script
*Habit*	Had developed a habit of not coming to campus
*HomeOfficeEnv*	Home office had better environment (technology, table, chair, etc.)
*HybridGood*	Hybrid worked well even for interactivity
*Language*	Student or lecturer struggled with German
*Lazy*	I was too lazy (explicitly stated)
*LecHallEnv*	Lecture-hall environment (noise, tables, chairs, crowdedness, etc.)
*MedDisab*	Medical reasons or disability (surgery, depression, ADHD, etc.)
*MilCivServ*	Had to serve in the military or alternative civil service
*MultCampus*	Due to the multiple campus locations and their distance
*NoDiff*	Made no difference how I attended (explicitly stated)
*NoHelpExercise*	Lecture did not help with exercices
*NoInterest*	I was not interested in the topic (explicitly stated)
*NoLive*	No live lectures were offered
*NonInteract*	Lecture was not interactive
*NotNew*	Material was not new to me
*NotUseful*	Lecture was not useful for me
*OffSem*	Exam would be in a later semester
*OtherPrio*	Had other priorities, was too busy
*PlayFaster*	Could play the recordings at higher speed
*PlayPauseRew*	Could pause or rewind the recordings
*PracTime*	Was more practical time-wise
*PrevSemRec*	Watched recordings from previous semesters
*Projects*	Was too busy doing project work
*RatherLive*	Would have rather come to live lecture
*Scheduling*	Scheduling did not work out
*ScreenShots*	Was able to make screenshots of the slides
*Sports*	Had sports (training, competitions)
*StudyAbroad*	Was studying abroad
*TooBoring*	Lecture was too boring
*TooDistract*	Got too easily distracted in live lecture on campus
*TooEarly*	Lecture was too early in the morning
*TooFast*	Lecturer was too fast
*TooSlow*	Lecturer was too slow
*Work*	Had to work (jobs, internships, assistantships, etc.)

**Fig. 9:**
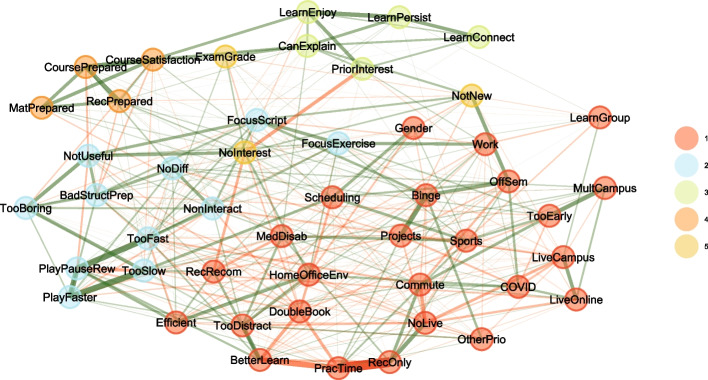
Exploratory cluster analysis for the categories in Table 4 that were mentioned more than 50 times, as well as course-relevant attributes (see Table 1), only considering *RecOnly* ≥ 50% or *LiveCampus* < 50% (see Table 2 and Fig. 8)

Figure 9 shows an exploratory cluster analysis for the categories that were mentioned more than 50 times (see Fig. 8), as well as the attributes in Table 1 that were relevant to the course (i.e., excluding attributes that pertain solely to the exam).

#### Circumstances and attendance choices

Cluster 1 includes external factors and interestingly also the attendance modes, suggesting that these choices are mostly connected to current circumstances. Here, however, the negatively correlated sub-cluster of *BetterLearn*, *DoubleBook*, and *PracTime* might be an unfortunate artifact of the survey design: students were only given a single choice of these pre-determined attributes, and in fact, many students wrote “all of the above” in the free-response text (which was then of course considered during the manual classification).

The strong negative correlation between *RecOnly* and *PracTime* is surprising: the choice to watch the recordings seems to be more than just independent of “being more practical time-wise,” but be made due to other factors (with having no live lectures to begin with (*NoLive*) being a trivial one). Surprisingly often, it was mentioned that the lecture-hall setting is distracting (*TooDistract*), and that thus it is better to learn outside of that setting (*BetterLearn*). It should be mentioned that the category *TooDistract* is linked to *MedDisab*; students did not feel comfortable or able to focus in the lecture hall. While most students did not specify their particular medical condition, several mentioned Attention-Deficit/Hyperactivity Disorder (ADHD) or depression:

“I just wasn’t doing so well.”

“Depression; when I had a good period, I could look at the records and catch up on the material I missed.”

“I have ADHD and find it very difficult to concentrate on the course on site. The distraction from other students is too great.”

The category *AloneCampus* did not make the 50-response cut, but while “depressed” may not have been used in the clinical sense in the following statement, there is a possibility that these attributes and categories feed on themselves and propagate each other:

“Because I felt depressed not to have friends to share my experiences with. Particularly because it was a difficult topic.”

—students do not come to lecture because they feel depressed that other students do not come to lecture.

The recommendation for other students to only watch the recordings (*RecRecom*) is linked to the perceived efficiency of the online media (*Efficient*) and *TooDistract*; 70.8% of the students who only watched the recordings for at least half of the lectures stated that they would recommend the same to others. Thus, most of these students who did so still seemed content with their sole reliance on recordings even after writing the exam.

*Efficient* was a large topic, frequently discussed all by itself:

“Time is a scarce resource! By participating online, you can make better use of the ‘dead time’ between lectures (empty spaces in the timetable) or the time over lunch and quickly gain 3 to 4 hours per day.”

The category *BetterLearn* was also connected to *Efficient*, which in turn was linked to the home-office environment (*HomeOfficeEnv*). For example, students frequently mentioned that in the lecture hall, the tables were too small to spread out materials while learning—other factors included lecture hall seats, which were distracting because they became uncomfortable after sitting for a long time. One student summarized the benefits of the home-office environment as follows:

“I like to watch live from home, because here I have multiple screens, a perfectly set up workstation, chair, table, ergonomic and designed for my needs—and no distraction from other people.”

Many of these home offices may have been furnished during the pandemic, and several students talked about having established their “home-office day,” which makes sense given the developments in the workplace they will eventually enter. In fact, COVID-19 still played a role in many respects, being associated with more *LiveOnline* instead of *LiveCampus* attendance. A typical remark was:

“The pandemic is not over yet! With little regard for students keeping their distance and the mask requirement lifted, I no longer felt able to protect my loved ones as well as I should—especially since long-term effects and LongCovid have not been well researched.”

Commutes may have gotten longer: students wrote that during the pandemic, they moved back in with their parents (also suggested by the positive link between *COVID* and *Commute*), particularly since living in Zurich is so expensive. Coupled with *TooEarly*, students rather watched live online:

“Home office eliminates the commute (almost 2 hours one way). The lecture was already at 8:15, which meant that I had to get up before 5:00 am if I wanted to see the lecture live on-site (I don’t have an apartment in Zurich, because it is extremely expensive and hard to get anything good).”

Having lecture halls spread across the city (*MultCampus*) did not help: students rather watched live online, particularly—once again—if the lecture was “too early in the morning.”

Other factors are studying abroad and still following lectures at the home university (*StudyAbroad*, which did not make the 50-response cut to appear in Fig. 9), or being involved in competitive sports (*Sports*), or worse, an unfortunate combination of these factors:

“I play competitive sports and have been abroad, and then injured and on crutches.”

Not really belonging into Cluster 1 seems to be the viewing choice *Binge*, i.e., watching several lectures back-to-back in a short amount of time—it seems to fit better into Cluster 2, discussed below. However, a strong external factor seems to be *Projects*, as several students explain:

“Design is so demanding that there is no time for lectures.”

“The semester project took up too much time, so that attending the lecture with adequate preparation and follow-up was not possible.”

Another reason for binge-watching are the delayed exams (*OffSemester*), as other students put it:

“The lecture was given during the winter semester while the exam is in the summer, and it didn’t make much sense for me to attend the lecture during the time it was given since I would have had to spend a long time during the summer trying to remember the contents.”

“It was one semester before, so by then I would have forgotten everything. I watched the lectures during the semester the exam took place in.”

Some students are even more cynical about this, particularly when the course deals with learning materials by heart:

“The course was offered one semester before the exam, and since no understanding is involved to pass, there was no point in attending the lecture. I watched the course before the exam and not one year before.”

A surprising observation that would make *Binge* part of Cluster 2 is the notion of coherence in learning mentioned by some students:

“I prefer to watch all lectures in 2 days instead of 14 weeks, because I see the connections better that way.”

—however, this particular concept did not clearly link up to any of the other categories; however, the average exam scores for binge-watchers was $5.0\pm 0.8$, which is slightly higher than average (significant at $p<0.05$).

#### Consumption choices and lecture attributes

Cluster 2 deals mostly with ways that recordings were consumed, depending on lecture attributes. Two of the choices seem obvious:

- If the lecture is perceived as too slow (*TooSlow*), students would watch the recording at a higher speed (*PlayFaster*). This is also connected to *TooBoring*.
  “The lecture, which is not very interesting, could be watched faster.”
  “I was able to watch the video at a rate of 1.25/1.5.”
  Students who watched lectures at a higher speed on the average scored $4.9\pm 0.9$, which is slightly higher than the overall average (significant at $p<0.05$).
- If the lecture is perceived as too fast (*TooFast*), students would pause or rewind the recording (*PlayPauseRew*). As one student puts it:
  “Way too fast-paced instruction and illegible writing. Once I lost the thread, I could not ’catch up’ as what was already written was very difficult to decipher. With the recording, I could pause and go back if needed, as the professor speaks along with what he writes to avoid deciphering. Also, much more convenient to understand the material since you’re not busy writing as fast as possible but can focus on the content.”
  Students who paused and reviewed on the average scored $4.8\pm 0.8$, that is, essentially the same as the overall average (no significant difference, $p\approx 0.5$).

Manipulating the playback speed does not necessarily lead to less time invested:

“If the professor told me something I didn’t understand, I could listen to it a third time. This is a big bonus for me. Furthermore, other areas that were already known could be shortened by speeding up the video. On average, I needed almost 2 hours per double lesson without a break, but I was able to get a lot out of this lesson.

More interesting are the connections to *NonInteract*—this has a stronger link to *TooFast* than to *TooBoring*. Interaction appears to be perceived not as a means to make lectures less boring, but as a means to digest the flood of information, which also explains the links to *FocusScript* and *FocusExercise*. The following comment is typical of the ensuing frustration:

“The instructor simply reads the slides without explanation. This is helpful for those who can’t read.”

“As a frontal class, this lecture was not so interactive that it needed a presence.”

Lectures characterized by students as badly structured or insufficiently prepared (*BadStructPrep*) are not only connected to there being no perceived difference to watching them online (*NoDiff*) or them simply being not useful (*NotUseful*), but also to *TooBoring* and attempts to compensate by playing some sections faster and rewinding others. It is important to note that we only asked students why they did *not* come to lectures—we thus would not have statements from the students who did find it worthwhile to come.

An unexpected link between *PlayFaster* and the ability to concentrate was revealed in several comments:

“At triple speed, it’s easier to watch the lectures, you get less distracted.”

“I was able to focus on the material better when I was following the recording at 1.5 times the normal speed, because otherwise I probably would have drifted off with my mind.”

The students who provided these quotes scored 5.0 and 4.75 on their exams, respectively. Overall, the students who made statements like these scored $4.8\pm 0.9$, which is essentially the same as the overall average (no significant difference, $p\approx 1$).

In addition, some students run the videos through cloud-based services that cut out pauses and stuttering, which some students directly hooked up to the university’s video portal through a browser plugin.

#### Remaining clusters

Cluster 3 corresponds to Cluster 2 in Figs. 6 and 7. The strongest link to reasons for not going to live lecture is through a rather obvious negative correlation between *PriorInterest* and *NoInterest*.

Cluster 4 is essentially the same as Cluster 1 in Figs. 6 and 7 except for the exam-specific items not considered here. It has no strong connection to reasons for not going to class, the strongest one being a negative correlation between *CoursePrepared* and *NotUseful*.

Cluster 5 contains *NoInterest*, *NotNew*, and *ExamGrade*. Intriguingly, the correlation between *NoInterest* and *ExamGrade* is positive—students can be successful on exams about topics they are not interested in, maybe because they pose little challenge. The correlation between *NotNew* and *ExamGrade* on the other hand is negative, possibly because the material is not new for students who already had to repeat the exam after failing it earlier (which would be suggested by the positive correlation of *NotNew* to *OffSem*). *NoInterest* is negatively correlated to *RecRecom*, suggesting that students, in retrospect, would not recommend only watching the recordings because they were not interested in the topic.

## Discussion

On the surface, the study seems to suggest that the mode of lecture attendance—attended in person, attended online, watched at normal speed, sped up or frequently paused, or even disregarded altogether—does not make a difference in exam grades.

On the one hand, that interpretation is too simplistic, since students were not randomly forced into particular attendance modes. They made their choices, and what is seen is the result of the choices that likely worked best for them. In fact, consciously and consequently making such choices appears important, since students who switched between attendance modes had statistically significantly worse exam grades—by a fraction of points, that is. This result is not new, it is compatible with hundreds of older studies (Russell, 1999) and more recent studies under the same (post-)pandemic conditions (Kortemeyer et al., 2022).

While this survey is much larger and spans more subjects than the similar survey of physics courses at an American university (Kortemeyer et al., 2022), results are comparable, including the somewhat puzzling result that live-lecture attendance on campus would be the dominating attribute when it comes to similarities between survey responses within courses that had recordings.

The responses in this survey were markedly different from those on student well-being at ETH Zurich at the height of the pandemic; while those responses frequently remarked on loneliness, lack of daily structure, depression, anxiety, a feeling of resignation, and lack of motivation, the responses in this survey (with the important exception of *MedDisab*) were noticeable more goal-oriented, pragmatic, determined, and frequently ambitious. It is a testimony to the resilience and flexibility of students, faculty, and staff, that apparently new arrangements were found and considered choices were made. Following the theory of self-determination, autonomy (the feeling of having a choice) is one of the three powerful basic needs that underlie human growth and development and therefore learning (Ryan & Deci, 2000)—post-COVID-19 gave learners more choices than ever before. Self-regulation, self-organization, and flexibility directly impact satisfaction with online offerings (Scheel et al., 2022; Turan et al., 2022). Instructors could foster the self-regulation that is needed to manage this autonomy by providing more opportunities for formative assessment (Nicol & Macfarlane-Dick, 2006; Clark, 2012), which may reduce attrition (Sitzmann & Ely, 2010). In this context, it would also have been intriguing to find out if overall exam performance increased or decreased compared to pre-pandemic years, however, exam grades are invariably “curved,” i.e., their grading scale adjusted depending on the outcome, so this absolute comparison between years is not possible.

On the other hand, the survey made clear that these choices were not unrestricted. External circumstances, some voluntarily engaged with, like doing sports, others forced upon the students, like medical conditions or the aftermath of COVID-19, limited their choices, potentially away from what might have been the optimal learning scenario for the individual. Still, apparently, not much harm was done.

Some attendance choices away from live lecture may be attributed to the policies of institutions:

- Putting heavy emphasis on project work without making room in the academic calendar appears to influence priorities that students are setting during the semester.
- Having exams possibly more than one semester after the course leads to students being concerned about forgetting the material anyway by the time the exam comes, and thus postponing watching the lectures until later.
- Allowing or even relying on students being able to enroll in more than one lecture at the same time in different places invariably leads to them not attending at least one of those.

Based on this and other studies, with the possible exception of interactive-engagement courses found here and in other studies (e.g. Jensen et al., 2022), mastery of the content does not depend on the chosen mode of lecture attendance—this is less surprising when considering that this choice, with some external constraints, was made by the students themselves likely based on what they assumed works best for them. There are factors that would make live-attendance the superior mode of learning; Interactivity and activating learning techniques are a proven value-add over one-way frontal instruction and, by extension, simply watching recordings, when it comes to mastering content (Hake, 1998; Hofer et al., 2018), and we found significant indicators to this effect. As *CanExplain* was most closely correlated with *ExamGrade*, peer instruction (Crouch & Mazur, 2001; Vickrey et al., 2015; Riegler, 2019) would likely both foster learning and incentivize on-site attendance. The link between interactive-engagement and lecture attendance would warrant an additional study, which explicitly considers instructional strategies in addition to lecture-attendance modes.

Of course, content is not all that universities are called to convey—there is the “hidden curriculum” (Semper & Blasco, 2018) of seemingly untestable competencies and socialization into an academic environment. It has been argued that several of the elements of this hidden curriculum—learning to learn, learning the profession, learning to be an expert, and learning the game—could also be accomplished through distance education (Anderson, 2001), yet, the responses to our survey suggest that many of the students simply focus on efficiency and exam preparation. Believing in the importance of cross-disciplinary competencies gained through immersion in the academic environment, signals might be sent by increasingly considering alternative forms of examinations, for example group or collaborative exams (Stearns, 1996; Hodges, 2004; Wieman et al., 2014; Jang et al., 2017).

This focus on efficiency is particularly evident when it comes to watching recordings at a faster speed, which many learners see as a way to absorb the most content in the least amount of time. In this context, the connection between video comprehension and playback speed is intriguing; while previous research suggests that comprehension might suffer due to higher playback speeds (Ness et al., 2021), or at best remain unchanged (Lang et al., 2020), the student comments suggest that it would increase because their attention would not wander. Also intriguing are statements that it would be better for their learning to watch lectures back-to-back in a short amount of time than on a weekly base (*Binge*)—this actually may work well in the short run by learning just enough quickly enough to reproduce it on the exam (“bulimic learning”), but contradicts almost everything known and confirmed about spaced repetition and longterm retention of knowledge (Ebbinghaus, 1885; Murre & Dros, 2015). Related to this is the notion of worrying about forgetting the material if the exam is more than a semester later, and thus not even bothering to learn it until the phase right before the exam; this runs afoul of the ideal of a curriculum where knowledge is built up and constructed over time, that is, a curriculum where one course builds upon the next—yet, from the point of view of the learner, this is simply the most efficient way within the current system.

## Limitations

The study was conducted a technical university with mostly traditional lecture-style teaching, and results may not transfer well to discussion or seminar-style humanities courses. In international comparison, the university has a peculiar exam system, where exams are separated from the courses, both administratively and in time. Responses were collected in exam context and may thus be strongly focussed on exam performance rather than learning experiences.

## Conclusion

In a study of over 17,000 survey responses associated with high-stake exams at a large technical university, it was found that students are—for the most part—making conscious choices about how to attend or view lectures: on-site, online live, or from recordings. The apparent influence of these choices on grades is statistically significant but minimal, where especially the students who were going back-and-forth between attendance modes did slightly worse, and students who had above-average on-site attendance had a slightly higher probability of receiving an above-average grade. There is, however, no straightforward relationship along the lines of “the more students only watch recordings, the worse they do on exams.” However, it needs to be kept in mind that each student worked—or attempted to work—in the way that worked best for them; this was not a randomized experiment.

We found indication that interactive-engagement courses (using clicker-usage as proxy for this property) led to a significant effect of live-lecture attendance on exam performance for students in the lower performance quartile. In other words, for low-achieving students, frequent live attendance of interactive-engagement courses is associated with higher exam grades.

Instead, besides the students’ estimation of their grade, the strongest correlation to the actual exam grade existed between their feeling that they could explain the topics to other students. Associated strategies such as peer instruction are certainly most easily carried out on-campus, and above-average on-site attendance was associated with above-average studying in groups. Above-average on-site attendance was also associated with above-average satisfaction with the course, enjoyment of learning, and interest in the subject, however, no causality in either direction can be implied.

There were several external circumstances that limited the free choices of the students or nudged them away from on-site lectures. If universities want to encourage students coming back to campus, this should likely not happen by taking choices away, but by incentivizing on-site lecture attendance and removing some systemic hurdles to active participation. Methods like peer instruction and more interactivity through technology-enabled formative assessment might make on-site attendance more worthwhile, compared to watching the recording of one-way instruction.

The conveyance of subject-matter content is not the only teaching mission of higher education, and increased off-campus or even asynchronous lecture attendance might come at the expense of the “hidden curriculum” of cross-disciplinary competencies and socialization into academia. Making this mission more explicit might be another means to influence student choices.

## Data Availability

As per human-subject research approval, raw data from this survey is only available upon revision of the protocol.
